# The complete mitochondrial genome and phylogenetic position of the critically endangered Trinidad Piping Guan, *Pipile pipile* synonym *Aburria pipile* (Aves: Galliformes)

**DOI:** 10.1080/23802359.2016.1219622

**Published:** 2016-09-04

**Authors:** Amelia A. Grass, Charlotte Hosie, Ian L. McDowall

**Affiliations:** Department of Biological Sciences, University of Chester, Chester, UK

**Keywords:** Mitochondrial, *Pipile*, *Aburria*, Pawi, Cracidae, Galliformes

## Abstract

The complete mitochondrial genome of the Critically Endangered Trinidad Piping Guan, *Pipile pipile* (Jacquin 1784) synonym *Aburria pipile* was sequenced for the first time in this study. The genome is 16,665 bp in length with overall base compositions of 30.1, 23.7, 32.3 and 13.9% for A, T, C, and G, respectively. Structurally, the *P. pipile* mitogenome is comparable to that of other Galliformes, thereby demonstrating typical avian gene organization. The mitogenome was subsequently used to produce a revised phylogenetic placement of *P. pipile* within the Galliforme order, positioning the *Pipile* genus basal within the Cracidae family. It is further envisaged that this novel genomic data will contribute to a wider understanding of genetic relationships within the genus *Pipile* and the analysis of the evolutionary relationships of the Galliforme order in a wider avian context.

## Mitogenome announcement

The Trinidad Piping Guan *Pipile pipile* is a Critically Endangered Cracid (Galliforme) endemic to the island of Trinidad, with an estimated population of less than 200 individuals (Hayes et al. 2009a). The population has been in ongoing decline largely due to anthropogenic pressures (Hayes et al. 2009a, 2009b). The IUCN and BirdLife International recognize Trinidad Piping Guan as a species of concern, classified as Critically Endangered, protected under Appendix I of the Convention on the International Trade in Endangered Species (BirdLife International 2015; CITES 2015; IUCN 2015). This mitochondrial genome represents the novel use of genetic data sourced from contemporary Trinidad Piping Guans from the wild on the island of Trinidad.

Samples were sourced from wild Trinidad Piping Guans (*P. pipile*) in the Matura Forest Reserve, Northern Range, Trinidad. *P. pipile* mitochondrial genomes were amplified from the feathers of live birds and liver tissue from a nonviable embryo.

The complete mitochondrial genome of *P. pipile,* sequenced for both heavy and light strands, was 16,665 bp ±2 bp in length (Genbank: KU221051, KU221052, KU221053), 13 protein-coding genes, 2 rRNAs, and 22 tRNAs were identified demonstrating organization consistent with Galliforme mito-genomes. Base composition of the three complete genomes demonstrate an A + T bias (53.8%), where overall base composition of the genome were as follows; A (30.1%), T (23.7%), C (32.3%), and G (13.9%).

All compositional features of the genome were consistent between samples, with length variation occurring due to indels in the hypervariable sections of the control region. The majority of genes are encoded on the heavy strand, however, the ND6 gene and eight tRNA genes are light strand encoded, as in other Galliformes. The majority of genes are contiguous within the sequence, however, gene overlaps occur in four locations; tRNA^Gln^ and tRNA^Met^, tRNA^Cys^ and tRNA^Tyr^, ATP8 and ATP6, and ND4L and ND4. Additionally, noncoding intergenic spacers of variable length (1–13nts) occur between some genes. The additional nontranslated nucleotide at position 174 of the ND3 gene was observed consistent with the observation of this additional nucleotide in other Galliformes (Mindell et al. 1998).

**Figure 1.**
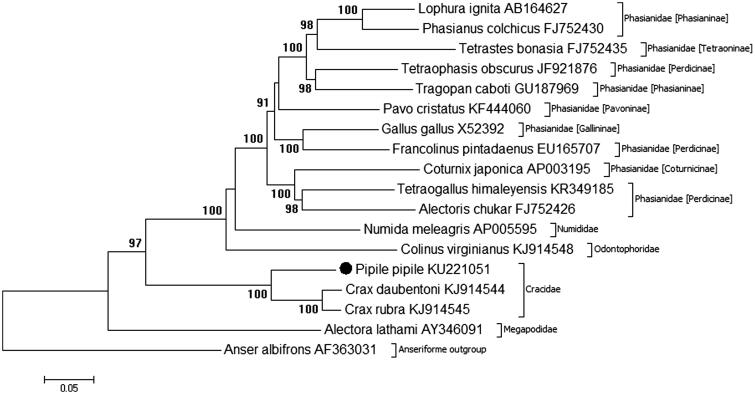
Phylogenetic relationships of *Pipile pipile* within the Galliforme order, inferred from whole mitochondrial genomes. Phylogenetic tree constructed using complete mitogenomes from Genbank in MEGA 7.0 using the Maximum-Likelihood method with 1000 bootstrap replicates. Genbank accession numbers are given after the species name, bootstrap support is given at each node.

Phylogenetic analysis of the mitochondrial genome of *P. pipile* and other Galliforme species is shown in Figure 1, constructed using MEGA7.0 (http://www.megasoftware.net/) (Kumar et al. 2016). The *Pipile* genus resolves basally within the Cracidae order of the Galliforme phylogenetic tree, with a high degree of bootstrap support (100%). Position of the Cracidae family within the Galliforme tree is consistent with initial mitogenome analysis (*Crax* sp.) in previous research (Meiklejohn et al. 2014). Basal divergence of the *Pipile* genus in relation to the *Crax* is consistent with individual mitochondrial gene analysis illustrated by Crowe et al. (2006), however, full resolution of the Cracidae family is restricted by a lack of complete mitochondrial genome information for Cracid species. The complete mitogenome of the Trinidad Piping Guan, *Pipile pipile*, is used for the first time in phylogenetic analysis, providing essential molecular data and evolutionary information for further analysis of both the *Pipile* genus and the Cracidae family.

## References

[CIT0001] Birdlife International 2015 BirdLife International Species Information Pipile pipile [Internet]. [cited 2015 Oct 10]. Available from: http://www.birdlife.org/datazone/speciesfactsheet.php?id =87.

[CIT0002] CITES. 2015 CITES Species Database [Internet]. [cited 2015 Oct 10]. Available from: http://speciesplus.net/#/taxon_concepts/7109/legal.

[CIT0003] Crowe TM, Bowie RCK, Bloomer P, Mandiwana TG, Hedderson TAJ, Randi E, Pereira SL, Wakeling J. 2006 Phylogenetics, biogeography and classification of, and character evolution in, gamebirds (Aves: Galliformes): effects of character exclusion, data partitioning and missing data. Cladistics. 22:495–532. 10.1111/j.1096-0031.2006.00120.x 34892896

[CIT0004] Hayes F, Sanasie B, Samad I. 2009a Status and conservation of the critically endangered Trinidad Piping-Guan Aburria pipile. Endanger Species Res. 7:77–84. [Internet]. [cited 2012 May 16]. Available from: http://www.int-res.com/abstracts/esr/v7/n1/p77-84/.

[CIT0005] Hayes F, Shameerudeen C, Sanasie B, Hayes B, Ramjohn C, Lucas F. 2009b Ecology and behaviour of the critically endangered Trinidad Piping-Guan Aburria pipile. Endanger Species Res. 6:223–229.

[CIT0006] IUCN. 2015 The IUCN Red List of Threatened Species. Red List [Internet]. [cited 2015 Oct 10]. Available from: http://www.iucnredlist.org/details/22678401/0.

[CIT0007] Kumar S, Stecher G, Tamura K. 2016 MEGA7: molecular evolutionary genetics analysis version 7.0 for bigger datasets. Mol Biol Evol 33:1870–1874. 27004904 10.1093/molbev/msw054 PMC8210823

[CIT0008] Meiklejohn KA, Danielson MJ, Faircloth BC, Glenn TC, Braun EL, Kimball RT. 2014 Incongruence among different mitochondrial regions: a case study using complete mitogenomes. Mol Phylogenet Evol. 78:314–323. 24929245 10.1016/j.ympev.2014.06.003

[CIT0009] Mindell DP, Sorenson MD, Dimcheff DE. 1998 An extra nucleotide is not translated in mitochondrial ND3 of some birds and turtles. Mol Biol Evol. 15:1568–1571. 12572620 10.1093/oxfordjournals.molbev.a025884

